# Editorial: SARS-CoV-2: implications for maternal-fetal-infant and perinatal mortality, morbidity, pregnancy outcomes and well-being

**DOI:** 10.3389/fped.2024.1375501

**Published:** 2024-02-08

**Authors:** Cheryl K. Walker, Balaji Govindaswami

**Affiliations:** ^1^ Department of Obstetrics and Gynecology, University of California-Davis, Davis, CA, United States; ^2^ The UC Davis MIND Institute, University of California, Davis, Sacramento, CA, United States; ^3^ Health Care Services, Valley Health Foundation, San Jose, CA, United States

**Keywords:** SARS-CoV-2, pregnancy outcomes infectious/epidemiology: infant outcomes infectious/epidemiology, premature birth/epidemiology, COVID-19 vaccination, post-acute COVID-19 syndrome

**Editorial on the Research Topic** SARS-CoV-2: implications for maternal-fetal-infant and perinatal mortality, morbidity, pregnancy outcomes and well-being

## Introduction

**Figure 1:**
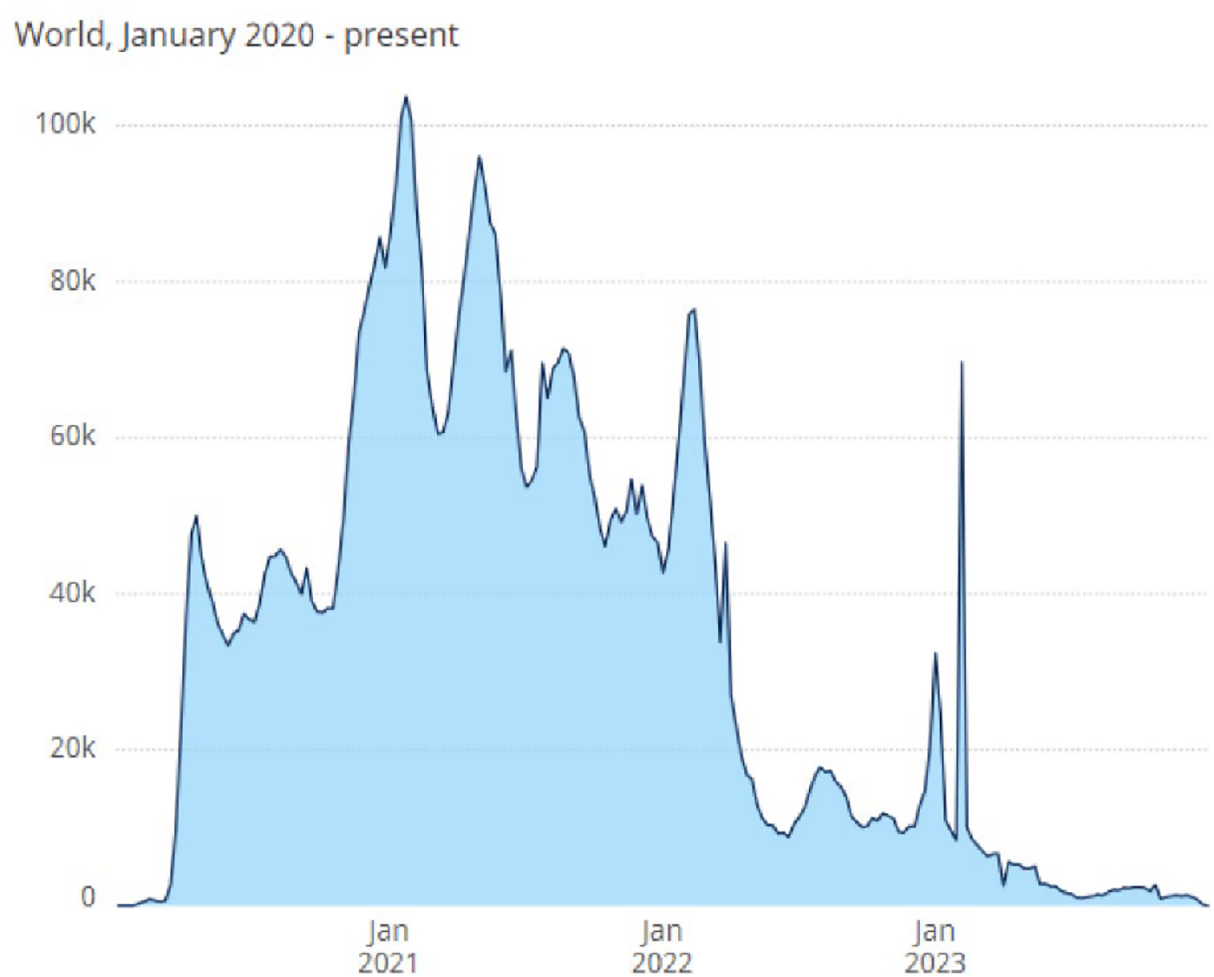
Total COVID-19 deaths reported to WHO (weekly). The World Health Organization (WHO) reports weekly deaths attributable to 3 COVID-19 infection worldwide (2).

On the fourth anniversary of the report of unusual pneumonia cases later identified as severe acute respiratory syndrome coronavirus 2 (SARS-CoV-2), the causal agent of Coronavirus Disease 2019 (COVID-19) (1), it is instructive to review what has been learned about the impact of this emerging global disease on the health and wellness of pregnant individuals, neonates, infants, and children. By the end of 2023, nearly 7 million COVID-19 deaths had been reported to the World Health Organization (WHO) (Figure 1) (2).

### Maternal effects and adverse pregnancy outcomes

Pregnant persons who contract COVID-19 are at increased risk for morbidity, intensive care unit admission, mechanical ventilation, and mortality compared with nonpregnant women (3–5) and those with diabetes mellitus, hypertension, and cardiovascular disease face greater severity of infection and adverse outcomes (6). SARS-CoV-2 is a multisystem disorder with particular affinity for neurological, immune and cardiovascular systems (7). COVID-19 in pregnancy increases risk for hypertensive disorders (8, 9). A study in this edition reported increased incidence of maternal chronic hypertension during the pandemic that linked to higher neonatal intensive care unit (NICU) admissions (Jegatheesan et al.). Affected populations were largely publicly insured individuals of color, accentuating existing obstetric health disparities. Hypertension in pregnancy predisposes to cardiovascular disease risk in the mother (10), intrauterine growth restriction, and programming of long-term cardiovascular (11) and neurodevelopmental health (12).

The impact of COVID-19 on preterm birth rates is complicated. Large cohort studies in international populations provided clear evidence that pregnant persons with symptomatic COVID-19 had significantly higher risk for preterm birth and NICU admission (3, 13, 14). Findings from temporal studies comparing rates before and after the onset of COVID-19 yielded mixed results likely reflecting other environmental influences. Lower preterm birth in multiple gestations in one German perinatal center was explained in part by restricted physical activity during lockdown (15). An analysis of 52 million births in 26 countries documented small decreases in preterm birth in the first 3 months of the pandemic lockdown, perhaps resulting from lower infection acquisition due to restricted social movement, better air quality from less traffic, and/or decrease in obstetric interventions for fetal wellbeing; only in Brazil was a concomitant increase in stillbirth noted (16). Several publications cited changing potency of circulating viral variants to explain fluctuating levels of infection acquisition and adverse perinatal outcomes over time (17–19). Others suggested that rising maternal immunity through prior infection or vaccination reduced infection incidence and complications over time (20, 21). The two studies included in this issue found no impact of COVID-19 on preterm birth rates (Rodriguez et al., Lorenzi et al.), which reinforces that infection risk is not randomly distributed in populations or over time and that combining data over several years may have diluted subtle time-sensitive effects.

### Infant morbidity/mortality and long-term population health

Newborn COVID-19 is rarely the result of vertical transmission and more commonly is acquired through contact with family members, healthcare workers, and visitors. Most cases are asymptomatic or mildly symptomatic (22). Two descriptive studies in this edition report mild clinical courses for COVID-19 infected neonates in Chinese study populations (Yang et al., Dai et al.). Also in this compendium is a review of dermatologic manifestations of COVID that is particularly useful in infants in whom case identification may be complicated (Young).

More serious infant and childhood manifestations are rare, with a retrospective cohort study from China in this edition reporting a 1.8% incidence of seizures in children aged 6 months to 3 years (Xu et al.). We also include a case series describing four children with moderate-to-severe neonatal hepatitis following omicron infection which cautions that clinicians monitor liver function during recovery (Wang et al.).

Importantly, the provisional infant mortality rate for the United States rose 3% from 2021 to 2022, the first year-to-year increase in two decades (23). The rise involved two leading causes of death: maternal complications and bacterial sepsis. While these data are preliminary and the underlying causes are likely to be multifactorial, COVID-19 may be a driver for the observed increase in infant mortality. The full impact of the pandemic on worldwide excess mortality has been estimated to exceed 300 deaths per 100,000 (24).

Long-term outcomes are being studied in children with fetal exposure to COVID-19. There is growing evidence that *in utero* exposure is associated with adverse neurodevelopmental sequelae, particularly in males (25, 26). Serious concerns reported in this edition involve a Brazilian birth cohort in which fetal COVID-19 exposure was associated with cerebral deep white matter changes suggesting zonal impairment of myelin content at 6 months adjusted age (Alves de Araujo et al.). These findings build on an established literature associating maternal infection, with fever and exaggerated immune response, with neurodevelopmental impairment including autism (27, 28).

Post-acute sequelae of COVID-19 infection (PASC) or Long COVID includes a broad set of persistent symptoms following infection. In a meta-analysis of 40 studies with 12,424 children, the pooled prevalence of Long COVID was 23.36% (29). A cohort study of 659,286 children with confirmed SARS-CoV-2 measured the incidence proportion of at least one feature of PASC was 41.9% in the COVID-positive group and 38.2% in those negative for COVID-19, for a difference of 3.7% (30). Increased rates were associated with acute illness severity, young age, and medical complexity. In adults, myalgic encephalomyelitis/chronic fatigue syndrome (ME/CFS), post-exertional malaise, memory loss and neurocognitive impairment are amongst the most common and debilitating Long COVID symptoms (31, 32). Systemic features of PASC often include viral persistence, chronic inflammation, hypercoagulability, and autonomic dysfunction (33, 34).

### Mechanistic insights into disease

Cytokines are essential regulators of the immune response that mediate protective inflammation. Early studies suggest that some individuals respond to COVID-19 with exuberant proinflammatory cytokine proliferation, with interferon-gamma (IFN-γ), Interleukin-1 beta (IL-1β), and IL-6 most implicated, particularly in severe cases (35, 36). Two contributions in this edition evaluated cord blood for evidence of COVID-19 vaccine or infection-induced immune and inflammatory biomarker elevation. One reported higher cord blood levels of cortisol, critical to fetal and neonatal anti-inflammatory activities, in pregnancies exposed to SARS-CoV-2 but did not find elevation in acute phase reactants (Mendenhall et al.). The other found no increase in cord blood cytokine levels (Jain et al.). Neither finding was unexpected, as cytokines have relatively short lives, and both studies had lags between maternal infection and sample collection.

Underlying molecular mechanisms have been hypothesized in adult PASC. Mitochondrial dysfunction, involving impaired cellular energy production with redox imbalance and oxidative stress, has been implicated in the etiology of Long COVID (37) and the efficacy of coenzyme Q10 (CoQ10) supplementation is being investigated as a therapeutic strategy (38). Reduction in serotonin levels through viral and immunological processes in PASC appears to impair vagal nerve, hippocampal responses and memory and targeted interventions are under investigation (39). An elegant longitudinal cohort study explored the pathophysiology of Long-COVID post-exertional malaise and found that exercise caused immediate skeletal muscle alterations, including reduction in mitochondrial enzyme activity, increased accumulation of amyloid-containing deposits, blunted T-cell response, and severe tissue damage (40). The implications for all these findings in children are unclear but profoundly concerning.

### Implications for health care services

Perinatal care practices evolved rapidly during lockdown in response to broad concerns for patient and provider safety Most face-to-face visits were replaced by remote monitoring and telehealth. Investigators are evaluating the adequacy of these health service modifications retrospectively. Three studies in this edition addressed the issue, with reassuring findings. One identified a slight delay in the timing of mid-pregnancy anatomy ultrasound scans during the pandemic that was unlikely to be clinically significant (Handley et al.). Another reported an increase in NICU admissions for hypoxic-ischemic encephalopathy (HIE) evaluation related to maternal hypertension but found no difference in HIE diagnosis or treatment (Song et al.). A final study demonstrated that there was no change in NICU discharge orders for maternal milk, though insured mothers were twice as likely to be providing milk perhaps due to the benefits of telework options not available to uninsured individuals (Boudreau et al.).

## Conclusion

This edition of *Frontiers in Pediatrics* adds to the existing SARS-CoV-2 literature in important ways. While serious pregnancy adverse outcomes appear to be attenuating due to preventive and treatment measures, maternal infection may induce cardiovascular and immune changes with profound implications for the mother and fetus. *In utero* exposure may lead to a form of Long COVID that induces brain changes and neurodevelopmental consequences. Evidence continues to reassure that most neonatal and pediatric COVID-19 infections are mild, but clinicians must remain vigilant for rare more serious manifestations and the potential for Long COVID. Investigation of PASC and its underlying pathophysiology and molecular mechanisms in children is a high priority, as is the impact of telehealth on pregnant individuals, infants, and children in the endemic stage of COVID-19. Vaccination strategies must creatively target pregnant persons and infants 6 months of age and older (41, 42). Finally, given the disproportionate impact of the pandemic on underrepresented communities already predisposed to excess perinatal morbidity and mortality, health officials must re-focus resources to optimize perinatal care quality through attention to the social determinates that place these populations at unacceptably enhanced risk.

## References

[1] Cucinotta D Vanelli M. WHO declares COVID-19 a pandemic. Acta Biomed. (2020) 91(1):157–60. 10.23750/abm.v91i19397 32191675 PMC7569573

[2] Organization WH. WHO COVID-19 dashboard. (2024).

[3] Allotey J Stallings E Bonet M Yap M Chatterjee S Kew T Clinical manifestations, risk factors, and maternal and perinatal outcomes of coronavirus disease 2019 in pregnancy: living systematic review and meta-analysis. Br Med J. (2020) 370:m3320. 10.1136/bmj.m3320 32873575 PMC7459193

[4] Zambrano LD Ellington S Strid P Galang RR Oduyebo T Tong VT Update: characteristics of symptomatic women of reproductive age with laboratory-confirmed SARS-CoV-2 infection by pregnancy status—United States, January 22–October 3, 2020. MMWR Morb Mortal Wkly Rep. (2020) 69(44):1641–7. 10.15585/mmwr.mm6944e3 33151921 PMC7643892

[5] Briller JE Aggarwal NR Davis MB Hameed AB Malhame I Mahmoud Z Cardiovascular complications of pregnancy-associated COVID-19 infections. JACC Adv. (2022) 1(3):100057. 10.1016/j.jacadv.2022.100057 35967591 PMC9364954

[6] Smith ER Oakley E Grandner GW Rukundo G Farooq F Ferguson K Clinical risk factors of adverse outcomes among women with COVID-19 in the pregnancy and postpartum period: a sequential, prospective meta-analysis. Am J Obstet Gynecol. (2023) 228(2):161–77. 10.1016/j.ajog.2022.08.038 36027953 PMC9398561

[7] Chatzis DG Magounaki K Pantazopoulos I Bhaskar SMM. COVID-19 and the cardiovascular system-current knowledge and future perspectives. World J Clin Cases. (2022) 10(27):9602–10. 10.12998/wjcc.v10.i27.9602 36186205 PMC9516937

[8] Baracy M J Afzal F Szpunar SM Tremp M Grace K Liovas M Coronavirus disease 2019 (COVID-19) and the risk of hypertensive disorders of pregnancy: a retrospective cohort study. Hypertens Pregnancy. (2021) 40(3):226–35. 10.1080/10641955.2021.1965621 34428127

[9] Kuriloff M Patel E Mueller A Dada T Duncan C Arnolds D COVID-19 and obstetric outcomes: a single-center retrospective experience in a predominantly black population. J Matern Fetal Neonatal Med. (2023) 36(1):2196364. 10.1080/14767058.2023.2196364 37005011

[10] Poon LC Nguyen-Hoang L Smith GN Bergman L O’Brien P Hod M Hypertensive disorders of pregnancy and long-term cardiovascular health: FIGO best practice advice. Int J Gynaecol Obstet. (2023) 160(Suppl 1):22–34. 10.1002/ijgo.14540 36635079

[11] Bhunu B Riccio I Intapad S. Insights into the mechanisms of fetal growth restriction-induced programming of hypertension. Integr Blood Press Control. (2021) 14:141–52. 10.2147/IBPC.S312868 34675650 PMC8517636

[12] Scime NV Hetherington E Tomfohr-Madsen L Nettel-Aguirre A Chaput KH Tough SC. Hypertensive disorders in pregnancy and child development at 36 months in the all our families prospective cohort study. PLoS One. (2021) 16(12):e0260590. 10.1371/journal.pone.0260590 34852012 PMC8635344

[13] Gholami R Borumandnia N Kalhori E Taheri M Khodakarami N. The impact of COVID-19 pandemic on pregnancy outcome. BMC Pregnancy Childbirth. (2023) 23(1):811. 10.1186/s12884-023-06098-z 37993814 PMC10664522

[14] Smith ER Oakley E Grandner GW Ferguson K Farooq F Afshar Y Adverse maternal, fetal, and newborn outcomes among pregnant women with SARS-CoV-2 infection: an individual participant data meta-analysis. BMJ Glob Health. (2023) 8(1):1–19. 10.1136/bmjgh-2022-009495 PMC9895919 36646475

[15] Delius M Kolben T Nussbaum C Bogner-Flatz V Delius A Hahn L Changes in the rate of preterm infants during the COVID-19 pandemic lockdown period-data from a large tertiary German university center. Arch Gynecol Obstet. (2023):1–9. 10.1007/s00404-023-07048-y 37231277 PMC10212226

[16] Calvert C Brockway MM Zoega H Miller JE Been JV Amegah AK Changes in preterm birth and stillbirth during COVID-19 lockdowns in 26 countries. Nat Hum Behav. (2023) 7(4):529–44. 10.1038/s41562-023-01522-y 36849590 PMC10129868

[17] Carlson J Simeone RM Ellington S Galang R DeSisto CL Fleming-Dutra K Pre-delta, delta, and omicron periods of the coronavirus disease 2019 (COVID-19) pandemic and health outcomes during delivery hospitalization. Obstet Gynecol. (2024) 143(1):131–8. 10.1097/AOG.0000000000005449 37917932 PMC10949122

[18] DeSisto CL Wallace B Simeone RM Polen K Ko JY Meaney-Delman D Risk for stillbirth among women with and without COVID-19 at delivery hospitalization—United States, March 2020–September 2021. MMWR Morb Mortal Wkly Rep. (2021) 70(47):1640–5. 10.15585/mmwr.mm7047e1 34818318 PMC8612508

[19] Favre G Maisonneuve E Pomar L Daire C Poncelet C Quibel T Maternal and perinatal outcomes following pre-delta, delta, and omicron SARS-CoV-2 variants infection among unvaccinated pregnant women in France and Switzerland: a prospective cohort study using the COVI-PREG registry. Lancet Reg Health Eur. (2023) 26:100569. 10.1016/j.lanepe.2022.100569 36628358 PMC9815480

[20] Torche F Nobles J. Vaccination, immunity, and the changing impact of COVID-19 on infant health. Proc Natl Acad Sci U S A. (2023) 120(49):e2311573120. 10.1073/pnas.2311573120 38011548 PMC10710065

[21] Kim H Kim HS Kim HM Kim MJ Kwon KT Cha HH Impact of vaccination and the omicron variant on COVID-19 severity in pregnant women. Am J Infect Control. (2023) 51(3):351–3. 10.1016/j.ajic.2022.07.023 35921943 PMC9339152

[22] De Luca D Vauloup-Fellous C Benachi A Vivanti A. Transmission of SARS-CoV-2 from mother to fetus or neonate: what to know and what to do? Semin Fetal Neonatal Med. (2023) 28(1):101429. 10.1016/j.siny.2023.101429 36935314 PMC10010052

[23] Ely D Driscoll AK. Infant mortality in the United States: provisional data from the 2022 period linked birth/infant death file. NVSS vital statistics rapid release. National Center for Health Statistics. (2023. Report #33. 39680705

[24] Collaborators C-EM. Estimating excess mortality due to the COVID-19 pandemic: a systematic analysis of COVID-19-related mortality, 2020–21. Lancet. (2022) 399(10334):1513–36. 10.1016/S0140-6736(21)02796-3 35279232 PMC8912932

[25] Edlow AG Castro VM Shook LL Kaimal AJ Perlis RH. Neurodevelopmental outcomes at 1 year in infants of mothers who tested positive for SARS-CoV-2 during pregnancy. JAMA Netw Open. (2022) 5(6):e2215787. 10.1001/jamanetworkopen.2022.15787 35679048 PMC9185175

[26] Edlow AG Castro VM Shook LL Haneuse S Kaimal AJ Perlis RH. Sex-specific neurodevelopmental outcomes among offspring of mothers with SARS-CoV-2 infection during pregnancy. JAMA Netw Open. (2023) 6(3):e234415. 10.1001/jamanetworkopen.2023.4415 36951861 PMC10037162

[27] Antoun S Ellul P Peyre H Rosenzwajg M Gressens P Klatzmann D Fever during pregnancy as a risk factor for neurodevelopmental disorders: results from a systematic review and meta-analysis. Mol Autism. (2021) 12(1):60. 10.1186/s13229-021-00464-4 34537069 PMC8449704

[28] Meltzer A Van de Water J. The role of the immune system in autism spectrum disorder. Neuropsychopharmacology. (2017) 42(1):284–98. 10.1038/npp.2016.158 27534269 PMC5143489

[29] Zheng YB Zeng N Yuan K Tian SS Yang YB Gao N Prevalence and risk factor for long COVID in children and adolescents: a meta-analysis and systematic review. J Infect Public Health. (2023) 16(5):660–72. 10.1016/j.jiph.2023.03.005 36931142 PMC9990879

[30] Rao S Lee GM Razzaghi H Lorman V Mejias A Pajor NM Clinical features and burden of postacute sequelae of SARS-CoV-2 infection in children and adolescents. JAMA Pediatr. (2022) 176(10):1000–9. 10.1001/jamapediatrics.2022.2800 35994282 PMC9396470

[31] Ceban F Ling S Lui LMW Lee Y Gill H Teopiz KM Fatigue and cognitive impairment in post-COVID-19 syndrome: a systematic review and meta-analysis. Brain Behav Immun. (2022) 101:93–135. 10.1016/j.bbi.2021.12.020 34973396 PMC8715665

[32] Komaroff AL Lipkin WI. ME/CFS and long COVID share similar symptoms and biological abnormalities: road map to the literature. Front Med (Lausanne). (2023) 10:1187163. 10.3389/fmed.2023.1187163 37342500 PMC10278546

[33] Merad M Blish CA Sallusto F Iwasaki A. The immunology and immunopathology of COVID-19. Science. (2022) 375(6585):1122–7. 10.1126/science.abm8108 35271343 PMC12828912

[34] Fedorowski A Fanciulli A Raj SR Sheldon R Shibao CA Sutton R. Cardiovascular autonomic dysfunction in post-COVID-19 syndrome: a major health-care burden. Nat Rev Cardiol. (2024). 10.1038/s41569-023-00962-3 38163814

[35] Tanacan A Yazihan N Erol SA Anuk AT Yucel Yetiskin FD Biriken D The impact of COVID-19 infection on the cytokine profile of pregnant women: a prospective case-control study. Cytokine. (2021) 140:155431. 10.1016/j.cyto.2021.155431 33503581 PMC7810028

[36] Forrest AD Poliektov NE Easley KA Michopoulos V Ravi M Cheedarla N Characterization of the inflammatory response to COVID-19 illness in pregnancy. Cytokine. (2023) 170:156319. 10.1016/j.cyto.2023.156319 37544133

[37] Komaroff AL Lipkin WI. Insights from myalgic encephalomyelitis/chronic fatigue syndrome may help unravel the pathogenesis of postacute COVID-19 syndrome. Trends Mol Med. (2021) 27(9):895–906. 10.1016/j.molmed.2021.06.002 34175230 PMC8180841

[38] Mantle D Hargreaves IP Domingo JC Castro-Marrero J. Mitochondrial dysfunction and coenzyme Q10 supplementation in post-viral fatigue syndrome: an overview. Int J Mol Sci. (2024) 25(1). 10.3390/ijms25010574 38203745 PMC10779395

[39] Wong AC Devason AS Umana IC Cox TO Dohnalova L Litichevskiy L Serotonin reduction in post-acute sequelae of viral infection. Cell. (2023) 186(22):4851–67.e20. 10.1016/j.cell.2023.09.013 37848036 PMC11227373

[40] Appelman B Charlton BT Goulding RP Kerkhoff TJ Breedveld EA Noort W Muscle abnormalities worsen after post-exertional malaise in long COVID. Nat Commun. (2024) 15(1):17. 10.1038/s41467-023-44432-3 38177128 PMC10766651

[41] COVID-19 Vaccines While Pregnant or Breastfeeding. Atlanta, GA: Centers for Disease Control and Prevention (2019). Available online at: https://www.cdc.gov/coronavirus/2019-ncov/vaccines/recommendations/pregnancy.html#:∼:text=CDC%20recommends%20everyone%20ages%206,become%20pregnant%20in%20the%20future (accessed January 11, 2024).

[42] 6 Things to Know About COVID-19 Vaccination for Children. Atlanta, GA: Centers for Disease Control and Prevention (2023).

